# A Biofuel Cell for Electricity Generation from Biomass-Derived Cellobiose

**DOI:** 10.3390/bios15100674

**Published:** 2025-10-07

**Authors:** Piyanut Pinyou, Peeranat Jatooratthawichot, Luciranon Sribrahma, Salila Pengthaisong, Chamaipon Beagbandee, Kantapat Chansaenpak, Vincent Blay, James R. Ketudat Cairns

**Affiliations:** 1 School of Chemistry, Institute of Science, Suranaree University of Technology, 111 University Ave., Muang, Nakhon Ratchasima 30000, Thailand; pj.cbsfa@gmail.com (P.J.); m6500733@g.sut.ac.th (L.S.); salila@sut.ac.th (S.P.); moobiotech22@gmail.com (C.B.); 2 Center of Biomolecular Structure, Function and Application, Suranaree University of Technology, 111 University Ave., Suranari, Nakhon Ratchasima 30000, Thailand; 3 National Nanotechnology Center, National Science and Technology Development Agency Thailand Science Park, Pathum Thani 12120, Thailand; kantapat.cha@nanotec.or.th; 4 Department of Microbiology and Environmental Toxicology, University of California at Santa Cruz, Santa Cruz, CA 95064, USA; 5 Laboratory of Biochemistry, Chulabhorn Research Institute, 54 Kamphaeng Phet 6 Rd, Laksi, Bangkok 10210, Thailand

**Keywords:** biofuel cell, β-glucosidase, glucose oxidase, cellobiose, biomass

## Abstract

We have developed a new bioanode based on a cascade of reactions catalyzed by two enzymes. A glassy carbon electrode is modified with β-glucosidase and glucose oxidase enzymes entrapped within an osmium redox polymer. Cellobiose, the fuel for the anode, is hydrolyzed by β-glucosidase (*Tx*GH116), yielding two molecules of D-glucose. Glucose is then oxidized by glucose oxidase (GOx) into δ-gluconolactone and produces electrons that are transferred to the electrode mediated by osmium redox polymer. We investigated the kinetic parameters of both enzymes at different temperatures. For GOx, the effect of enzyme loading and enzyme/polymer ratio were also optimized. The proposed bioanode is coupled to a biocathode based on horseradish peroxidase (HRP) in which H_2_O_2_, the oxidant, is reduced. We investigated the performance of the biofuel cell on cellobiose and sugarcane hydrolysates subjected to different pretreatments. Alkaline pretreatments of biomass were found to be more effective than phosphoric acid pretreatment. Adding *Tx*GH116 β-glucosidase further enhanced current generation, even when commercial cellulase was used.

## 1. Introduction

The pursuit of sustainable and efficient energy solutions has intensified the interest in biofuel cells (BFCs), particularly enzymatic biofuel cells (EBFCs), which convert biochemical energy into electrical energy under mild, environmentally friendly conditions [1]. Multi-enzyme cascades have emerged as a powerful approach to enhance EBFC performance [2]. In these systems, multiple enzymes enable a reaction network that converts complex substrates into valuable products or energy carriers [3,4,5]. In EBFC systems, by enabling a more complete oxidation of fuels through sequential catalytic steps, multi-enzyme cascades offer significantly higher energy conversion efficiency and greater power density than single-enzyme systems [6,7]. For instance, Minteer’s group demonstrated the oxidation of glucose to carbon dioxide through a cascade of six enzymes, achieving the extraction of 24 electrons per glucose molecule. Large biomass-derived molecules, such as cellulose, glycogen, and starch, can also serve as fuels after enzymatic breakdown [8,9]. For example, hydrolases such as cellulases break down lignocellulosic materials into fermentable sugars [10], and oxidoreductases such as glucose oxidase (GOx) [10] or pyranose dehydrogenase (PDH) [11] can oxidize these sugars, releasing electrons that can be captured as electrical current. Recently, Wu et al. developed a biosensor for the real-time monitoring of sucrose uptake in plants. The EBFC utilized a carbon fiber anode modified with a three-enzyme cascade consisting of an invertase (hydrolase), a mutarotase (isomerase), and a glucose oxidase (oxidoreductase) [12]. Sequential multi-enzyme conversions over electrodes provide a direct path to transform abundant biomass into electrical energy [13], supporting the 7th Sustainable Development Goal—affordable and clean energy

β-Glucosidase (EC 3.2.1.21) plays a pivotal role in cellulose-based biomass degradation [13]. It catalyzes the hydrolysis of glucosides such as cellobiose into glucose [14,15]. Nearly all β-glucosidases hydrolyze glucosides by a double-displacement mechanism that leads to retention of chirality at the anomeric carbon [16,17]. Reducing the levels of cellobiose is also desirable as it can inhibit other enzymes used in biomass saccharification [18]. Enzyme cocktails containing β-glucosidase are used in bioethanol production [19], biorefineries [20], and emerging bioelectrochemical systems [11]. β-Glucosidases also have applications in food and beverage industries [16,21].

Although β-glucosidases are particularly abundant in plants, enzymes of microbial origin are often favored for their rapid degradation of cellulosic materials [22,23,24]. Researchers have also devoted significant efforts to engineering β-glucosidases with improved catalytic efficiency, thermostability, and glucose tolerance [25,26,27], and a wide variety of β-glucosidases are available. A GH116 β-glucosidase from the thermophile *Thermoanaerobacterium xylanolyticum*, *Tx*GH116, has a temperature optimum of 75 °C and a melting temperature of 81 °C [28]. *Tx*GH116 has a known structure, and the roles of catalytic and glucose-binding amino acids have been delineated by mutagenesis [28,29,30]. Catalytic residue mutants of *Tx*GH116 have proven useful for production of glucosylazides [31,32]. Although *Tx*GH116 is relatively sensitive to glucose inhibition with a competitive *K*_i_ of 4 mM, its stability and high catalytic rate on oligosaccharides make it attractive for applications, especially at low to moderate glucose concentrations. In addition, it shows much less inhibition by δ-gluconolactone, the product of GOx, than most β-glucosidases, making it appropriate for application in glucose fuel cells [25].

As a primary product of cellulose hydrolysis, cellobiose is a promising renewable fuel for biofuel cells (BFCs). In this work, we designed and evaluated a bioanode platform capable of efficiently converting cellobiose and cellulosic hydrolysates into electrical current through a two-step enzymatic process. In this system, β-glucosidase *Tx*GH116 catalyzes the hydrolysis of cellobiose into D-glucose, which is subsequently oxidized by GOx to δ-gluconolactone, with electrons transferred to the electrode via mediated electron transfer (MET) using an Osmium redox polymer. This configuration enables current generation from a renewable disaccharide as fuel. As proof of concept, the BFC system was evaluated using both cellobiose and hydrolysates derived from sugarcane leaves.

## 2. Materials and Methods

### 2.1. Reagents, Enzymes and Solutions

D-Glucose, glucose oxidase (GOx) from *Aspergillus niger* (≥65 U mg^−1^), and D-(+)-cellobiose were obtained from TCI Chemicals (Japan). Poly(ethylene glycol) diglycidyl ether (PEGDGE), horseradish peroxidase (HRP (≥174 U mg^−1^), 2,2′-azino-bis-(3-ethylbenzothiazoline-6-sulfonic acid) ammonium salt (AzBTS) and Nafion^TM^ 117 were purchased from Sigma-Aldrich (St. Louis, MO, USA). PVI-Os(bpy)_2_Cl_2_ was prepared according to the established protocol [33]. A bioanode electrode was modified on a surface of a 4 mm glassy carbon disk electrode (Rotating disk electrode, RDE, ALS, Japan). *Tx*GH116 was expressed and purified as previously described with a single immobilized metal affinity chromatography step [28].

### 2.2. ThCel6A Production and Purification

Based on the bacteria’s tolerance to high temperatures and alkaline pH, a *Thermobifida halotolerans* glycoside hydrolase family GH6 endoglucanase (NCBI Accession AHN09982, *Th*Cel6A) was selected. A gene optimized for expression in *Escherichia coli* encoding this protein without its N-terminal signal sequence (residues 31-443) and containing a stop codon (Figure S1) was synthesized and cloned into the *Nco*I and *Xho*I sites of pET32a by Gene Universal Corporation. The plasmid was used to transform *E. coli* strain BL21(DE3) and the protein produced in an 800 mL LB broth culture in a 2 L shake flask induced with 0.2 M IPTG for 18 h at 20 °C shaking at 200 rpm. The cells were centrifuged at 4000× *g* 20 min at 4 °C to remove the media and the cell pellet frozen at −80 °C, then thawed and lysed in 20 mL lysis buffer containing 50 mM sodium phosphate, pH 7.5, 0.2 mg/mL lysozyme, 1% Triton-X 100, 1 mM phenylmethylsulfonyl fluoride (PMSF), 1 mM 6-aminohexanoic acid, 1 mM benzamidine hydrochloride and 5 µg/mL DNase I for 30 min at 25 °C. The cell debris was removed by centrifugation at 12,000× *g* for 20 min at 4 °C. The protein was purified over an immobilized metal affinity chromatography resin column (GE Healthcare) bound with CoCl_2_ and equilibrated in 150 mM NaCl, 50 mM sodium phosphate, pH 7.5 (eq buffer). After loading the column, it was washed with 5 column volumes each eq buffer, 5 mM imidazole in eq buffer, and 10 mM imidazole in eq buffer, then eluted with 250 mM imidazole in eq buffer. The fractions containing *Th*Cel6A were pooled and concentrated and the buffer exchanged with 150 mM NaCl, 20 mM Tris, pH 8.0 (enzyme storage buffer) in a centrifugal filter, MWCO 30,000 (Merck Millipore, Darmstadt, Germany). The protein concentration was determined by measuring absorbance at 280 nm on a Nano drop spectrophotometer (Thermo Fisher Scientific, Wilmington, DE, USA, NanoDrop™ 2000/2000c). Sodium dodecyl sulfate-polyacryamide gel electrophoresis (SDS-PAGE) was performed on 12% acrylamide gels to determine the purity and molecular mass of the protein which was visualized by Coomassie Blue R-250 staining.

### 2.3. Enzymatic Characterization of ThCel6A

The recombinant *Th*Cel6A was tested for hydrolysis of the soluble β-glucan substrates carboxymethyl cellulose (CMC) and barley β-glucan by the 3,5-dinitrosalicylic acid (DNS) assay for reducing sugars [34]. The enzyme (2 µg) was assayed on 0.5% CMC or barley β-glucan in 50 mM sodium acetate, pH 5.5, at temperatures ranging from 20 to 80 °C for 24 h, followed by boiling 5 min to stop the reactions and DNS assay of released sugars. For the pH optimum, the enzyme was assayed in McIlvaine citrate/phosphate universal buffers over a range of pH 3.5 to pH 9.0 in 0.5 unit intervals at 50 °C for 30 min. To evaluate the temperature stability, the enzyme was incubated at 40, 50, 55 or 60 °C for 1 to 24 h in 50 mM sodium acetate, pH 5.5, then assayed for hydrolysis of 0.5% CMC at 50 °C for 30 min; then the reaction stopped by boiling and the reducing sugar released assayed by the DNS assay. The products of hydrolysis of CMC and barley β-glucan were also assessed by stopping the reactions at various time points by boiling, then evaluating the supernatant by thin-layer chromatography (TLC) on silica gel 60 plates (Merck KGaA, Darmstadt, Germany). The plates were developed in butanol:water:acetic acid (4:2:2, *v*/*v*/*v*). The products were visualized by spreading 10% sulfuric acid in ethanol on the plate and heating to develop gray spots at carbohydrates.

### 2.4. Enzymatic Hydrolysis of Insoluble Biomass

To prepare biomass, sugarcane leaf was dried in an oven at 65 °C until it was completely dried. Then, it was cut into approximately 1 cm long pieces and was milled in a PULVERISETTE 16 cross beater mill (Fritsch, Germany) and passed through a 500 μm sieve. For alkaline pretreatments, 2 g of milled leaf was pretreated with 50 mL of 2% (*w*/*v*) sodium carbonate or sodium hydroxide that was autoclaved at 121 °C 20 min. The pretreated sugarcane leaf was rinsed with distilled water until it reached a pH of about 7 (3–5 times), then dried in the oven at 65 °C overnight. Phosphoric acid pretreatment was performed as described by Wood et al. [35] and Zhang et al. [36].

The pretreated sugarcane leaf biomass was digested in either commercial cellulase (Novozyme Cellic Celltec2) or *Th*Cel6A. 10 mL of a 1% (w/v) suspension of pretreated sugarcane leaf powder in 50 mM sodium acetate buffer pH 5.5 was digested overnight with 1% (*w*/*v*) cellulase or 3 mg *Th*Cel6A to produce the hydrolysates for testing in the bioanode.

### 2.5. Bioanode and Biocathode Preparation

A bioanode was prepared by drop casting 7 μL of a mixture containing 40 μg PVI-Os(bpy)_2_Cl_2_, 20 μg GOx and 21 μg PEGDGE in DI water on the electrode surface of a 4 mm glassy carbon electrode. The modified electrodes were left to dry in the air at room temperature and subsequently kept in the refrigerator at 4 °C overnight. Prior to measurement, the modified electrodes were rinsed with citrate phosphate buffer pH 5.5 to remove the weakly adsorbed components from the electrode surface. A biocathode was prepared by drop-casting 20 µL of 1-pyrenebutyric acid N-hydroxysuccinimide ester (PBSE) at a concentration of 4 mg/mL in dimethylformamide (DMF) onto the surface of a 9 mm graphene-coated polyimide electrode. The electrode was incubated at room temperature for 30 min to allow π–π stacking interactions between PBSE and the graphene surface. Following incubation, the electrode was gently rinsed with DMF to remove any unbound PBSE and then dried under ambient conditions. Subsequently, 100 µg of 10 mg/mL (10 μL) horseradish peroxidase (HRP) in phosphate buffer was drop-cast onto the PBSE-modified electrode surface and allowed to dry at room temperature. After drying, 2 µL of 5% (*w*/*v*) Nafion solution was drop-cast over the enzyme layer to immobilize and protect the biomolecule. The fully assembled biocathode was then stored at 4 °C overnight to ensure proper stabilization of the enzyme and Nafion matrix.

### 2.6. Electrochemical Characterization

The electrochemical characterization and optimization of both the bioanode and biocathode were performed with a PalmSens potentiostat (PalmSens, Utrecht, The Netherlands). A conventional three-electrode setup was employed, comprising a platinum sheet (1 × 1 cm) as the counter electrode and an Ag/AgCl 3 M KCl reference electrode (Italsens, Italy).

For bioanode experiments, citrate buffer (pH 5.5) was used as the supporting electrolyte. The catalytic performance of the GOx-modified bioanode was assessed by chronoamperometry, monitoring the oxidation current of glucose at a constant applied potential of 0.28 V versus Ag/AgCl 3 M KCl. Prior to measurements, the electrolyte was purged with argon (Air Liquide, Thailand) for at least 30 min to remove dissolved oxygen, and all electrochemical measurements were subsequently carried out under an argon atmosphere.

Temperature control during measurements was achieved with a water-jacketed electrochemical cell equipped with a temperature controller (IKA-Werke GmbH & CO. KG, Staufen im Breisgau, Germany, model ICC basic), ensuring the electrolyte was equilibrated to the desired experimental temperature. For biocathode studies, measurements were conducted under ambient air.

### 2.7. Biofuel Cell Performance

The performance of the cellobiose/H_2_O_2_ enzymatic biofuel cell (BFC) was evaluated in a two-compartment electrochemical cell separated by a Nafion™ 117 membrane. The glucose oxidase (GOx) bioanode was connected to the working electrode and immersed in 20 mL of 50 mM sodium acetate buffer pH 5.5 containing 30 mM cellobiose or 1% (*w*/*v*) sugarcane leaf hydrolysate supplemented with 93 μg of *Tx*GH116.

The horseradish peroxidase (HRP) biocathode was connected to the combined counter and reference electrodes and immersed in 25 mL of electrolyte solution containing 10 mM H_2_O_2_ and 1.0 mM AzBTS. Prior to electrochemical measurements, the anolyte was left to incubate for 15 min after the addition of *Tx*GH116.

Open-circuit voltage (OCV) was recorded until a stable voltage output from the BFC was obtained. Subsequently, multistep amperometry was performed by applying a series of potential steps ranging from 0.60 V to 0.010 V vs. the OCV of the BFC. During operation, the bioanode compartment was maintained under an argon-saturated atmosphere, while the biocathode was exposed to an air-saturated environment. The electrolyte temperature was kept constant at 40 °C throughout the experiment.

## 3. Results and Discussion

### 3.1. GOx-Modified Anode Characterization and Optimization

**Figure 1:**
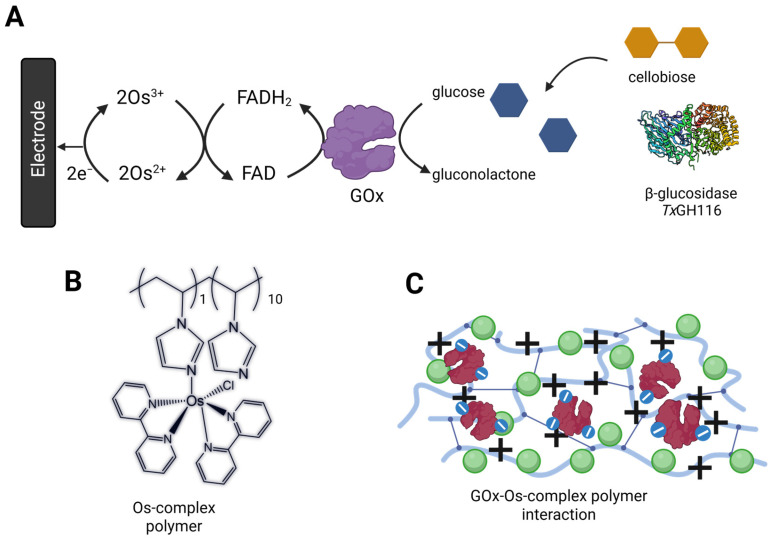
**Bioanode construction.** (**A**) Overall reaction from cascade enzymatic reaction based on β-glucosidase (*Tx*GH116) and GOx. (**B**) Structure of PVI-Os(bpy)_2_Cl_2_. (**C**) The electrostatic interaction forms an adduct between the negatively charged GOx and positively charged PVI-Os(bpy)_2_Cl_2_. Panel A and C were created in BioRender. Pinyou, P. (2025) https://BioRender.com/fbytq6b.

The bioanode was designed to utilize cellobiose as its substrate. This disaccharide is hydrolyzed by β-glucosidase into two molecules of D-glucose, as illustrated in Figure 1. Since β-glucosidase is a hydrolase and does not participate in redox reactions, the electrochemical activity of the bioanode relies on the GOx-catalyzed oxidation of glucose. Therefore, the bioanode was initially optimized using glucose as the substrate. The electrode was modified by entrapping GOx within a poly(1-vinylimidazole)-osmium complex [PVI–Os(bpy)_2_Cl_2_] matrix.

Redox polymers bearing osmium (Os) complexes have been widely employed for the co-immobilization with GOx, enabling electron transfer from the enzymatic oxidation of glucose to the electrode surface via a mediated electron transfer (MET) pathway through an electron-hopping mechanism [37,38,39]. In addition to facilitating efficient electron shuttling, redox polymers support the entrapment of large quantities of redox enzymes, forming multilayered enzyme assemblies. The resulting redox hydrogel provides a three-dimensional network that promotes rapid substrate diffusion and enhances charge transport across the film [40].

#### 3.1.1. Optimization of GOx to Redox Polymer Mass Ratio

Optimizing the relative amounts of redox polymer and GOx is crucial to ensure efficient electron transfer within the bioanode. A sufficient number of redox relay units, provided by the osmium complex in the polymer is essential to electrically wire the redox-active site of GOx to the electrode surface, thereby enabling effective mediated electron transfer.

**Figure 2:**
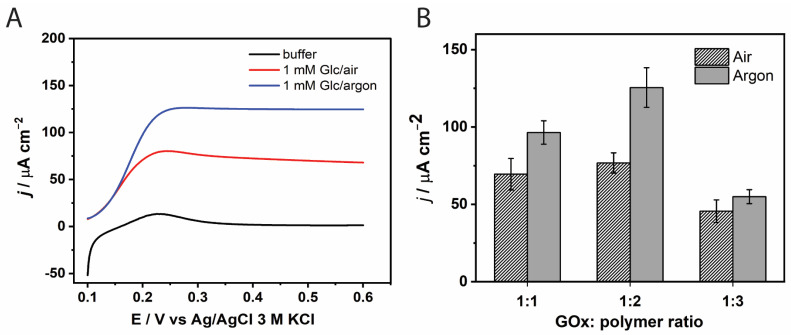
Effect of GOx: redox polymer weight ratio on the current density for glucose oxidation of GOx/PVI-Os(bpy)_2_Cl_2_/GCE. (**A**) LSV voltammograms of the electrode modified with 1:2 GOx-to-polymer ratio recorded in the absence and presence of 1 mM glucose under air or argon saturated electrolyte. (**B**) The bar graph was plotted from the anodic current density of 1 mM glucose in citrate buffer pH 5.5 at the applied potential 0.28 V vs. Ag/AgCl 3 M KCl, rotation speed = 500 rpm at 30 °C.

The influence of the GOx-to-redox-polymer ratio on the current density was investigated under both oxygenated and deoxygenated (argon-saturated) conditions, as shown in Figure 2B. Previous studies have reported negative effects of oxygen on the current output of GOx-modified electrodes [41,42]. Two mechanisms have been proposed to explain this effect: (i) oxygen can react with the reduced form of GOx, producing hydrogen peroxide (H_2_O_2_), which may degrade the electrode components, and (ii) oxygen competes with the redox mediator in re-oxidizing GOx, thereby hindering efficient electron transfer [41]. Consistent with these reports, our results show that current densities measured in air-equilibrated electrolyte were significantly lower than those obtained in argon-saturated conditions (Figure 2).

Under argon-saturated conditions, the highest current density was observed at a GOx:PVI-Os(bpy)_2_Cl_2_ mass ratio of 1:2 (Figure 2B). This enhancement can be attributed to the optimal availability of osmium-based redox relays, which facilitates efficient electron transport via the electron-hopping mechanism. However, further increasing the amount of redox polymer (1:3) led to a marked decrease in current density. This reduction may be due to structural changes in the redox hydrogel in the acidic hydrolysate electrolyte. At low pH, the poly(1-vinylimidazole) (PVI) backbone undergoes substantial swelling due to protonation of the imidazole groups [43]. Mao et al. reported that a hydrogel based on a PVI-Os complex can swell to 3.5 times its dry thickness [44,45]. As a result, even a slight increase in polymer loading may increase film thickness and hinder glucose diffusion to the enzyme’s active site.

Based on these findings, a GOx-to-polymer mass ratio of 1:2 was selected, and all subsequent measurements of the GOx-modified electrode were performed in an argon-saturated electrolyte.

#### 3.1.2. Effect of GOx Loading Amount

**Figure 3:**
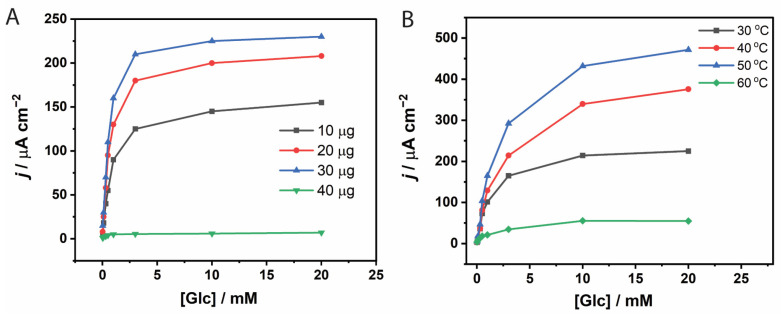
(**A**) Current densities of GOx-modified electrodes with different GOx loadings (10–40 μg) in citrate buffer pH 5.5 and 30 °C. (**B**) Current densities of the GOx-modified electrode at different temperatures (30–60 °C) and glucose concentrations (0.02–20 mM) at an applied potential of 0.28 V (vs Ag/AgCl 3 M KCl) under argon-saturated electrolyte and a stirring speed of 1000 rpm.

The GOx loading on the electrode surface was optimized by varying the enzyme amount from 10 to 40 µg (Figure 3A). An increase in current density was observed with increasing GOx from 10 to 30 µg. However, the enhancement in catalytic current between 20 and 30 µg was marginal. This plateau is attributed to the limited number of osmium redox centers in the polymer matrix, which restricts the ability to effectively shuttle electrons from the reduced form of GOx, thereby limiting the current output. Moreover, GOx is a non-conductive protein, and excessive loading may hinder glucose diffusion and further reduce catalytic efficiency. Considering both the current response and enzyme utilization, 20 µg of GOx was selected as the optimal loading for further studies.

#### 3.1.3. Effect of Temperature on Immobilized GOx Kinetics

Amperometric measurements with the GOx-modified electrode were conducted at electrolyte temperatures ranging from 30 °C to 60 °C. Increasing the temperature from 30 °C to 50 °C resulted in a steady rise in current density, reflecting faster enzymatic and electrochemical reaction rates (Figure 3B). However, a sharp decline in current density was observed when the temperature approached 60 °C, likely due to thermal instability or partial detachment of the GOx–polymer film from the electrode surface. Based on these findings, a temperature of 40 °C was selected for subsequent experiments to ensure sufficient catalytic activity while maintaining the structural integrity of the system.

### 3.2. Effect of Temperature on TxGH116 β-Glucosidase

**Figure 4:**
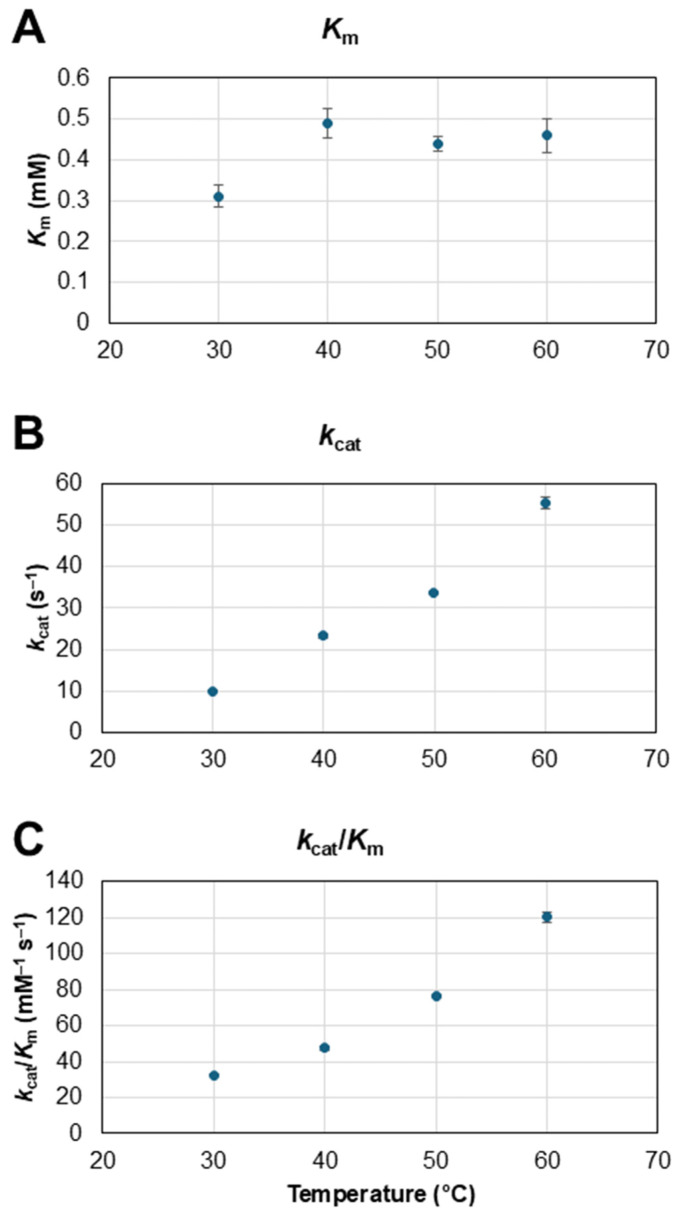
Effect of temperature on *Tx*GH116 kinetics for hydrolysis of cellobiose. Effect of temperature on apparent values for (**A**) *K*_m_, (**B**) *k*_cat_ and (**C**) *k*_cat_/*K*_m_ for *Tx*GH116 hydrolysis of cellobiose were assessed with 10 nM *Tx*GH116 in 50 mM sodium acetate, pH 5.5, in 20 min reactions at the designated temperatures.

Although *Tx*GH116 is stable at 60 °C [35], the effect of temperature on its cellobiose hydrolysis efficiency had not been previously assessed. Thus, the kinetics of cellobiose hydrolysis by *Tx*GH116 were assessed at temperatures over the range of 30 to 60 °C. As seen in Figure 4, the catalytic rate (*k*_cat_) and specificity constant (*k*_cat_/*K*_m_) for hydrolysis of cellobiose increased ca. 1.5–2-fold every 10-degree increase in this range, while *K*_m_ was relatively stable. The relevant parameter at the high cellobiose concentration in the electrode is *k*_cat_, which was 24 s^−1^ at the 40 °C temperature used for the electrode. If GOx electrode stability can be improved in the future, we could increase glucose production and subsequent power output by increasing the temperature.

### 3.3. Evaluation of Bi-Enzymatic GOx/β-Glucosidase Catalysis

While glucose in solution is an equilibrium mixture of 64% β-anomer and 36% α-anomer [46], the overall enzymatic hydrolysis of cellobiose by a retaining β-glucosidase is estimated to yield approximately 82% β-glucose, which will gradually equilibrate by mutarotation to the equilibrium composition. Because GOx selectively oxidizes β-D-glucose, we expect this out-of-equilibrium composition to be advantageous, as long as cellobiose does not inhibit GOx.

Before incorporating cellobiose as a substrate in the bi-enzymatic electrode system, we evaluated the potential inhibitory effect of cellobiose on the catalytic activity of GOx toward glucose oxidation. Amperometric measurements were conducted with the GOx-modified electrode in the presence of a constant 1 mM glucose concentration and cellobiose concentrations from 1.0 to 10 mM. As shown in Figure S2, the catalytic current remained essentially unchanged across the tested range of cellobiose concentrations, indicating that cellobiose does not interfere with or inhibit GOx activity under the given conditions.

**Figure 5:**
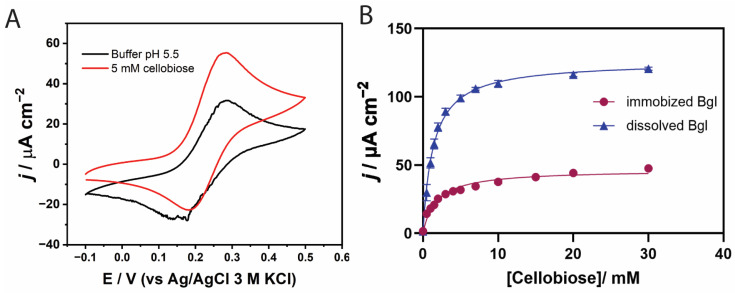
(**A**) Cyclic voltammograms of co-immobilized *Tx*GH116 Bgl-GOx/PVI-Os(bpy)_2_Cl_2_/GCE bioanode in the absence and presence of cellobiose 5 mM at a scan rate of 5 mV/s at 40 °C. (**B**) Current density profiles for systems with 10 μg immobilized GOx and 30 μg β-glucosidase in solution (blue), or 10 μg immobilized GOx and 30 μg immobilized β-glucosidase (purple), in argon−saturated citrate buffer pH 5.5 containing cellobiose (0.5–30 mM) at an applied potential of 0.28 V (vs Ag/AgCl 3 M KCl); rotation speed = 1000 rpm, T = 40 °C.

We investigated the electrochemical behavior of the enzyme-modified electrode using cyclic voltammetry. The cyclic voltammogram of the co-immobilized GOx/β-glucosidase in the redox hydrogel-modified bioanode is shown in Figure 5A. In the absence of cellobiose, the voltammogram exhibited quasi-reversible redox peaks corresponding to the Os^3+^/Os^2+^ redox couple, with a half-wave potential (E_1_/_2_) of 0.23 V, consistent with previous reports using the same polymer matrix [47]. Following the addition of cellobiose, the electrolyte was incubated with the modified electrode for 15 min to allow for hydrolysis by β-glucosidase. The resulting voltammogram displayed an increased anodic current associated with glucose oxidation at the electrode surface, accompanied by a cathodic peak shift of approximately 25 mV compared to the cellobiose-free condition. This observation indicates successful electron mediation between the FAD redox centers in GOx and the Os-complex in the polymer matrix.

The catalytic performance of the bi-enzymatic system under two different enzyme configurations. In the first setup, β-glucosidase was added directly to the electrolyte to catalyze the homogeneous hydrolysis of cellobiose, generating glucose in solution prior to its oxidation by the immobilized GOx. In the second configuration, both GOx and β-glucosidase were co-immobilized on the electrode. As shown in Figure 5B, the current density with β-glucosidase in solution (blue) was approximately three times higher than that in the co-immobilized system (purple) with a *j*_max_ of 120.5 mA cm^−2^. This enhancement can be attributed to improved substrate accessibility—cellobiose can interact with the active site of free β-glucosidase from all directions, whereas mass transport limitations may hinder access to the immobilized enzyme. In agreement with this, the freely diffusing β-glucosidase system exhibited a lower apparent Michaelis–Menten constant (*K*_m_^app^ = 1.37 mM) than the system with β-glucosidase immobilized (*K*_m_^app^ = 1.80 mM), indicating saturation at a lower cellobiose concentration. Based on these findings, the homogeneous β-glucosidase system was selected for subsequent experiments.

### 3.4. Current from Sugarcane Leaf Treated with Cellulase

To assess whether the fuel cell could be used to generate electricity from natural biomass rather than from the model substrate cellobiose, biomass was digested with commercial cellulase and the supernatant added to the biocathode with and without *Tx*GH116 β-glucosidase. Sugarcane leaves were selected as the agricultural waste to be evaluated, and three pretreatment methods were compared.

**Figure 6:**
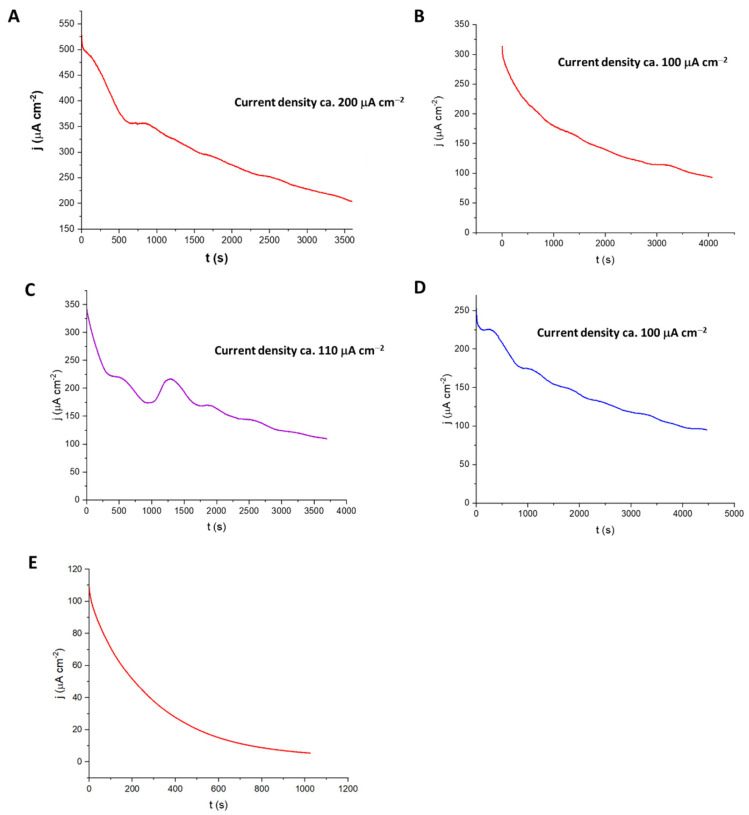
Current densities generated over time recorded at the GOx/PVI-Os(bpy)_2_Cl_2_/GCE bioanode at constant applied potential of 0.28 V vs Ag/AgCl 3 M KCl at 40 °C with sugarcane leaf (SCL) biomass substrate. (**A**) 1% SCL pretreated with sodium carbonate digested with 1% cellulase and 0.2 mg *Tx*GH116 β-glucosidase, the solution was stirred at 400 rpm. (**B**) 1% SCL pretreated with 1% cellulase alone. (**C**) 1% SCL pretreated with sodium hydroxide and digested with 1% cellulase and 0.2 mg *Tx*GH116 β-glucosidase. (**D**) 1% SCL pretreated with sodium hydroxide and digested with 1% cellulase alone. (**E**) 1% SCL pretreated with phosphoric acid and digested with 1% cellulase and 0.2 mg *Tx*GH116 β-glucosidase.

As seen in Figure 6, treatment with a 2 M sodium carbonate alkali led to the best output for this system. Adding *Tx*GH116 β-glucosidase provided an additional 100–200 μA cm^−2^ of output at each point over a 3500 s time course. A similar but less pronounced effect was seen with sodium hydroxide pretreatment of the sugarcane leaf biomass, although with lower current, while phosphoric acid-treated sugarcane leaf gave much lower current. The current density of ca 200 μA cm^−2^ for the system with commercial cellulase anve d added *Tx*GH116 suggests that this system could generate useful current from sugarcane leaf biomass.

### 3.5. Current from Sugarcane Leaf Treated with ThCel6A and TxGH116

The presence of a complex mixture of enzymes including β-glucosidase in the commercial cellulase mixture obscures the role of the β-glucosidase added to our bioanode. To better dissect the role of β-glucosidase, we generated a system with a single endoglucanase, *Th*Cel6A, which was previously shown to be thermostable and release cellobiose from CMC [48].

We expressed *Th*Cel6A with an additional thioredoxin fusion tag at the N-terminus and without the signal sequence at high levels and the purified (Figure S3) had high activity over a broad range of pH with a peak around pH 8–8.5 (Figure S4A), in line with the reported pH optimum [48], and 30% maximum activity at pH 5.5. The temperature optimum was 45 °C and it maintained nearly 80% maximal activity up to 65 °C (Figure S4B). The enzyme was stable up to 55 °C, with over 75% activity after 24 h (Figure S5). The good stability over the range of temperatures appropriate to our system and the broad pH range are favorable for use with the *Tx*GH116 β-glucosidase, whose optimal pH is 5.5.

The main *Th*Cel6A hydrolysis products after 24 h digestion of barley β-glucan, CMC, phosphoric acid swollen cellulose (PASC), base-treated cellulose, and base-pretreated rice straw and sugarcane leaves were di-, tri- and tetra-saccharides (Figures S6 and S7), which are appropriate for *Tx*GH116 β-glucosidase digestion. Glucose production was not seen except for a small amount from PASC, so use of *Th*Cel6A could make the contribution of *Tx*GH116 to the bioanode clearer than hydrolysis with commercial cellulase.

**Figure 7:**
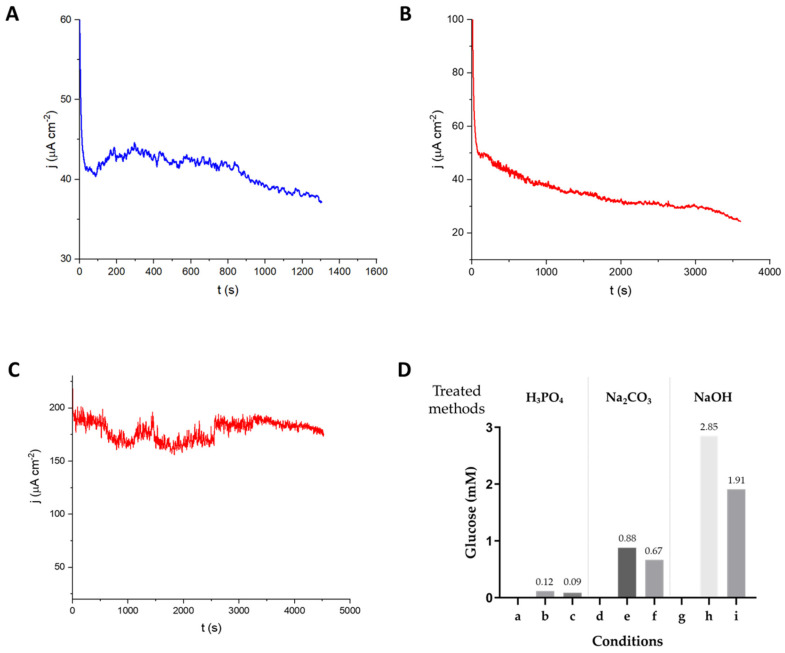
Current densities generated over time recorded at the GOx/PVI-Os(bpy)_2_Cl_2_/GCE bioanode at constant applied potential of 0.28 V vs Ag/AgCl 3 M KCl at 40 °C with sugarcane leaf biomass substrate in the solution under stirring at 400 rpm. (**A**) 1% SCL pretreated with phosphoric acid digested with 3 mg *Th*Cel6A and 0.2 mg *Tx*GH116 β-glucosidase. (**B**) 1% SCL pretreated with sodium carbonate digested with 3 mg *Th*Cel6A and 0.2 mg *Tx*GH116 β-glucosidase. (**C**) 1% SCL pretreated with sodium hydroxide digested with 3 mg *Th*Cel6A and 0.2 mg *Tx*GH116 β-glucosidase. (**D**) Glucose concentration determination by PGO assay from the hydrolysis of 1% SCL using *Th*Cel6A and β-glucosidase pretreated with different chemicals: a: 1% SCL treated with phosphoric acid; b: 1% SCL treated with phosphoric acid before current measurement; c: 1% SCL treated with phosphoric acid after current measurement; d: 1% SCL treated with Na_2_CO_3_; e: 1% SCL treated with Na_2_CO_3_ before current measurement; f: 1% SCL treated with Na_2_CO_3_ after current measurement; g: 1% SCL treated with NaOH; h: 1% SCL treated with NaOH before current measurement; i: 1% SCL treated with NaOH after current measurement.

Sugarcane leaf powders with three different pretreatments were tested in the bioanode after hydrolysis with *Th*Cel6A. The *Tx*GH116 β-glucosidase was also added to the digest to have glucose available at the start of the current measurement. As seen in Figure 7, pretreatment with phosphoric acid or sodium carbonate gave initial current densities around 40 µA cm^−2^ and maintained the current density above 30 µA cm^−2^ for more than 20 min. Notably, sodium hydroxide pretreatment provided a higher current density with around 180 µA cm^−2^ for 80 min (Figure 7C). Thus, the sodium hydroxide pretreatment gave about 4.5 to 6-fold higher current density than the other two. Over the time measured, the glucose concentration in the anolyte decreased by about one third (Figure 7D), since glucose was mainly released before the digestate was added to the bioanode. The current density was lower than that with hydrolysis with commercial cellulase, likely because the commercial cellulase includes many enzymes that can work together to more effectively break down complex biomass samples to release oligosaccharides for hydrolysis by β-glucosidase. However, the experiment with *Th*Cel6A demonstrated the effectiveness of *Tx*GH116 in converting oligosaccharides released from biomass to glucose for generation of electrons by glucose oxidase, since it was the only β-glucosidase in the mixture.

### 3.6. BFC Performance with Cellobiose and SCL Hydrolysates

**Figure 8:**
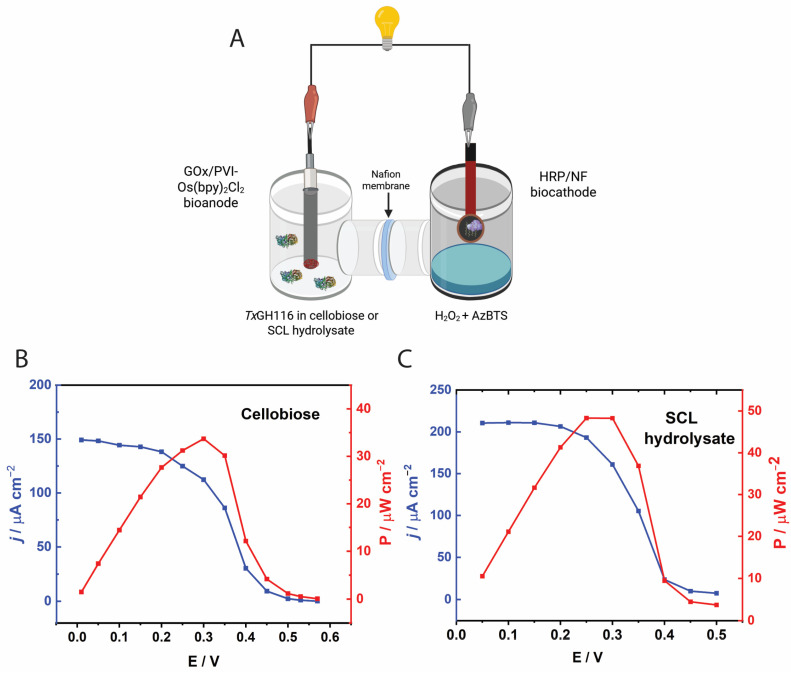
(**A**) Schematic diagram of the BFC assembled in a two-compartment electrochemical cell separated by a Nafion™ 117 membrane. Panel A was created in BioRender. Pinyou, P. (2025) https://BioRender.com/z3bxt05. **Panel B Cellobiose** (**B**) Polarization (blue) and power density (red) curves of the BFC comprised a GOx/PVI-[Os(bpy)_2_Cl]_2_-modified GCE bioanode operating in 30 mM cellobiose and 93 μg *Tx*GH116 β-glucosidase dissolved in citrate buffer (pH 5.5) under argon-saturated electrolyte and stirring at 400 rpm, and an HRP/NF/graphene biocathode immersed in 0.1 M phosphate buffer (pH 6.0) containing 0.15 M NaCl, 10 mM H_2_O_2_, and 1 mM AzBTS at 40 °C under air-equilibrated conditions. **Panel C Biomass-derived fuel (SCL hydrolysate)** (**C**) Polarization and power density curves of the BFC assembled in a two-compartment electrochemical cell consisting of GOx/PVI-[Os(bpy)_2_Cl]_2_-modified GCE bioanode in the 1% SCL pretreated with Na_2_CO_3_ hydrolysates hydrolyzed with the commercial cellulase at 40 °C under argon-saturated electrolyte and stirring at 400 rpm.

The performance of the biofuel cell (BFC) using cellobiose as the fuel was initially evaluated, as shown in Figure 8A. The polarization curve, obtained in a buffer containing 30 mM cellobiose after a 15 min incubation with *Tx*GH116 prior to electrochemical measurements, displayed a typical decrease in current density with increasing cell voltage, particularly near the open-circuit voltage (OCV). The observed OCV was approximately 0.57 V. The BFC achieved a maximum current density of around 150 µA cm^−2^. The corresponding power density curve peaked at approximately 0.30 V, yielding a maximum power output of about 33.7 µW cm^−2^. Although the need for a 15 min preincubation with β-glucosidase to build a reservoir of glucose may hinder continuous use, it may be less critical for use with commercial cellulase-treated biomass which already contains released glucose. Future improvement in β-glucosidase for more rapid hydrolysis can be used to achieve an appropriate steady-state level of glucose. Nonetheless, this experiment suggests that useful output from biomass is feasible.

Figure 8B presents the BFC performance when the bioanode was operated in 1% SCL hydrolysates derived from either commercial cellulase pretreated with Na_2_CO_3_. The OCV values obtained from both pretreated hydrolysates were approximately 0.50 V, which were lower than the OCV observed during operation with pure cellobiose (0.57 V). This reduction in OCV can be attributed to the increased complexity of the hydrolysate matrices, which may contain insoluble residues and non-fermentable components [49]. These residual substances likely contributed to elevated solution resistance and may have interfered with electron transfer at the bioanode. Additionally, certain compounds (phenolic derivatives) present in the hydrolysates could have exerted inhibitory effects on the enzymatic activity of β-glucosidase and GOx at the bioanode [50,51], thereby lowering the overall electrochemical potential of the BFC system. The BFC utilizing hydrolysate from commercial cellulase showed significantly enhanced performance, attaining a maximum current density of 210 µA cm^−2^ and a peak power density of 48 µW cm^−2^ at 0.30 V. This improvement reflects the efficient enzymatic saccharification of lignocellulosic biomass, resulting in higher glucose concentrations readily oxidized by the GOx-based bioanode.

## 4. Conclusions

In this study, we successfully demonstrated a cascade enzymatic catalysis strategy in the bioanode of a BFC, employing β-glucosidase and GOx for the hydrolysis and oxidation of cellobiose, a key product from lignocellulosic biomass. To enhance electron transfer efficiency between GOx and the electrode, an osmium-complex-modified polymer was utilized as a redox mediator, facilitating electrical communication between the FAD redox centers and the electrode surface.

Due to the relatively slow kinetics of β-glucosidase, a 15 min incubation was allowed before electrochemical measurements to allow for sufficient cleavage of cellobiose before electrooxidation of glucose catalyzed by GOx. Electrochemical analysis revealed that the bioanode configuration with GOx immobilized on the electrode and β-glucosidase dissolved in the electrolyte exhibited significantly higher current density compared to the co-immobilization of both enzymes on the electrode. This optimized bioanode system was successfully applied to generate current from hydrolysates of sugarcane leaf biomass pretreated under various conditions and with a cellulase cocktail or two β-glucosidases.

The application of this bioanode was further demonstrated by coupling it with a HRP-based biocathode for hydrogen peroxide reduction, with AzBTS serving as the redox mediator. When operated with cellulase hydrolysates of sugarcane leaf pretreated with sodium carbonate, the assembled BFC delivered a maximum power output of 50 µW cm^−2^ at an operating voltage of 0.4 V and an open-circuit voltage (OCV) of 0.5 V. Overall, this work highlights the potential of combining β-glucosidase- and GOx-catalyzed reactions for energy generation from biomass. Furthermore, the development and application of novel β-glucosidases with faster kinetics and high glucose tolerance through enzyme engineering may expand the performance of this system for future bioenergy applications.

## Data Availability

The data and materials that support the findings of this study are available from the corresponding authors upon reasonable request.

## Supplementary Materials

The following supporting information can be downloaded at: https://www.mdpi.com/article/10.3390/bios15100674/s1, Figure S1: Sequence of optimized gene encoding *Th*Cel6A and its corresponding protein sequence; Figure S2: Effect of cellobiose concentration on the GOx-bioanode; Figure S3: SDS-PAGE analysis of the purification fractions of *Th*Cel6A enzyme from recombinant expression; Figure S4: *Th*Cel6A endoglucanase activity dependence on pH and temperature; Figure S5: Thermal stability of *Th*Cel6A; Figure S6: Thin-layer chromatography of the hydrolysis product of *Th*Cel6A against Barley β D glucan and CMC; Figure S7: Thin-layer chromatography of the hydrolysis product of *Th*Cel6A against commercial and natural polysaccharide substrates with different incubation time.

## Abbreviations

AzBTS	2,2′-Azino-bis-(3-ethylbenzothiazoline-6-sulfonic acid) ammonium salt
BFCs	Biofuel cells
EBFCs	Enzymatic biofuel cells
Bgl	β-glucosidase
CMC	Carboxymethyl cellulose
DNS	3,5-dinitrosalicylic acid
GOx	Glucose oxidase
HRP	Horseradish peroxidase
MET	Mediated electron transfer
NF	Nafion
OCV	Open circuit voltage
PASC	Phosphoric Acid Swollen Cellulose
PGO	Peroxidase-Glucose Oxidase
SCL	Sugarcane leaf
SDS-PAGE	Sodium dodecyl sulfate-polyacryamide gel electrophoresis
TLC	Thin-layer chromatography
