# Supplementary material for: A Biofuel Cell for Electricity Generation from Biomass-Derived Cellobiose
Source: Biosensors (Basel). 2025 Oct 7;15(10):674. doi: 10.3390/bios15100674 (PMC12563716; doi:10.3390/bios15100674)
Supplement: Supplementary file 1 [file biosensors-15-00674-s001.zip › biosensors-3757566-supplementary.pdf]

biosensors

Supplemental Information

Biocatalytic System based on Bi-enzymatic beta-Glucosidase/Glucose Oxidase for a Cellobiose/H_2O_2 Biofuel Cell

Piyanut Pinyou [1,*,†], Peeranat Jatooratthawichot [1,2,†], Luciranon Sribrahma [1,2], Salila Pengthaisong [1,2], Chamaipon Beagbandee [1,2], Kantapat Chansaenpak [3], Vincent Blay [4,*] and James R. Ketudat Cairns [1,2,5,*]

[1] School of Chemistry, Institute of Science, Suranaree University of Technology, 111 University Ave., Muang, Nakhon Ratchasima 30000, Thailand; pj.cbsfa@gmail.com (P.J.); m6500733@g.sut.ac.th (L.S.); salila@sut.ac.th (S.P.); moobiotech22@gmail.com (C.B.)
[2] Center of Biomolecular Structure, Function and Application, Suranaree University of Technology, 111 University Ave., Suranari, Nakhon Ratchasima 30000, Thailand
[3] National Nanotechnology Center, National Science and Technology Development Agency Thailand Science Park, Pathum Thani 12120, Thailand; kantapat.cha@nanotec.or.th
[4] Department of Microbiology and Environmental Toxicology, University of California at Santa Cruz, Santa Cruz, CA 95064, USA
[5] Laboratory of Biochemistry, Chulabhorn Research Institute, 54 Kamphaeng Phet 6 Rd, Laksi, Bangkok 10210, Thailand
* Correspondence: piyanutp@sut.ac.th (P.P.); vroger@ucsc.edu (V.B.); cairns@sut.ac.th (J.R.K.C.)
† These authors contributed equally to this work.

Supplementary Contents:

Figure S1. Sequence of optimized gene encoding *Th*Cel6A and its corresponding protein sequence.
Figure S2. Effect of cellobiose concentration on the GOx-bioanode.
Figure S3. SDS-PAGE analysis of the purification fractions of *Th*Cel6A enzyme from recombinant expression.
Figure S4. *Th*Cel6A endoglucanase activity dependence on pH and temperature.
Figure S5. Thermal stability of *Th*Cel6A.
Figure S6 Thin-layer chromatography of the hydrolysis product of *Th*Cel6A against Barley β D glucan and CMC.
Figure S7 Thin-layer chromatography of the hydrolysis product of *Th*Cel6A against commercial and natural polysaccharide substrates with different incubation time.

```
32
ccatg gca aac gat agc ccg ttt tat gtg aat ccg gat atg gcc agt gca gaa tgg gtt cgt
   M   A   N   D   S   P   F   Y   V   N   P   D   M   A   S   A   E   W   V   R
aat aat ccg agc gat ccg cgc acc ccg gtt att cgt gat cgt gtt gca agt gtt ccg cag
 N   N   P   S   D   P   R   T   P   V   I   R   D   R   V   A   S   V   P   Q
gcc acc tgg ttt acc cag cat aat ccg ggc gaa att acc ggt cag gtg gat agc ctg gtg
 A   T   W   F   T   Q   H   N   P   G   E   I   T   G   Q   V   D   S   L   V
agt gcc gcc gcc agc gaa ggt aaa acc ccg att ctg gtt gtg tat aat gcc ccg ggt cgt
 S   A   A   A   S   E   G   K   T   P   I   L   V   V   Y   N   A   P   G   R
gat tgt ggc aat cat agc agc ggt ggc gca ccg agc cat gca gca tat cgt gat tgg gtg
 D   C   G   N   H   S   S   G   G   A   P   S   H   A   A   Y   R   D   W   V
gat gat ttt gca gcc ggt ctg aaa aat cgc ccg gca tat att gtg gtg gaa ccg gat ctg
 D   D   F   A   A   G   L   K   N   R   P   A   Y   I   V   V   E   P   D   L
att agt ctg atg gca agc tgc atg cag agc acc cag cag gaa gtt ctg cag acc ctg agc
 I   S   L   M   A   S   C   M   Q   S   T   Q   Q   E   V   L   Q   T   L   S
tat gca ggt aaa gcc ctg aaa gcc ggt agt agc cag gcc cgc gtg tat ttt gat gcc ggt
 Y   A   G   K   A   L   K   A   G   S   S   Q   A   R   V   Y   F   D   A   G
cat agc gcc tgg cat agc ccg agt cag atg gcc agc tgg ctg aat cag gca gat att agt
 H   S   A   W   H   S   P   S   Q   M   A   S   W   L   N   Q   A   D   I   S
aat agc gca gat ggc att gcc acc aat acc agt aat tat cgc tgg acc gca gat gaa gtt
 N   S   A   D   G   I   A   T   N   T   S   N   Y   R   W   T   A   D   E   V
gca tat gcc aaa gca gtt att gcc gca acc ggt gac agt agt ctg cgt gcc gtt att gat
 A   Y   A   K   A   V   I   A   A   T   G   D   S   S   L   R   A   V   I   D
acc agc cgt aat ggc aat ggt ccg gat ggt agc gaa tgg tgc gat ccg agc ggc cgc gca
 T   S   R   N   G   N   G   P   D   G   S   E   W   C   D   P   S   G   R   A
att ggt acc ccg agc acc acc aat acc ggc gat agc atg att gat gca ttt ctg tgg gtg
 I   G   T   P   S   T   T   N   T   G   D   S   M   I   D   A   F   L   W   V
aaa ctg ccg ggc gaa gcc gat ggc tgt att gcc agt gca ggt cag ttt gtt ccg gat gca
 K   L   P   G   E   A   D   G   C   I   A   S   A   G   Q   F   V   P   D   A
gcc tat gaa atg gca atg gca gcc ggt gac tat acc ccg gat ccg acc ccg gat cct gaa
 A   Y   E   M   A   M   A   A   G   D   Y   T   P   D   P   T   P   D   P   E
ccg acc ccg gac ccg gaa ccg acc cct gat ccg aat ccg gat ccg gat ggc gcc tgt acc
 P   T   P   D   P   E   P   T   P   D   P   N   P   D   P   D   G   A   C   T
gca acc tat agc att gcc aat gaa tgg gat aat ggt ttt cag ggc acc gtg acc gtg acc
 A   T   Y   S   I   A   N   E   W   D   N   G   F   Q   G   T   V   T   V   T
gca aat cag gcc att agt ggc tgg acc gtt acc tgg acc ttt acc gat ggt cag agt gtg
 A   N   Q   A   I   S   G   W   T   V   T   W   T   F   T   D   G   Q   S   V
agt aat gcc tgg aat gca acc gtt acc agc agc ggt agt agc gtg acc gcc agt gat gtt
 S   N   A   W   N   A   T   V   T   S   S   G   S   S   V   T   A   S   D   V
ggt tat aat ggt agc ctg ggt agt ggc gcc agt acc gaa ttt ggt ttt gtg gcc agc aaa
 G   Y   N   G   S   L   G   S   G   A   S   T   E   F   G   F   V   A   S   K
agt ggt gcc aat agc gtg ccg acc ctg acc tgc acc gca ggc tag ctc gag
 S   G   A   N   S   V   P   T   L   T   C   T   A   G   -   443
```

Figure S1. Sequence of optimized gene encoding *Th*Cel6A and its corresponding protein sequence. During expression, this was cloned into pET32a in frame with its N-terminal thioredoxin and His6 tags, thrombin site, S-tag and enterokinase site, which adds approximately 21 kDa to the 42.949 kDa predicted size of the mature protein, to give the expected mass of 64 kDa for the fusion protein. The starting position of the mature protein (Asn32) is marked with "32" for the residue position in the precursor protein and the end marked with 443, the residue position of the C-terminus. The stop codon is designated by a dash and restriction sites for cloning into pET32 are underlined.

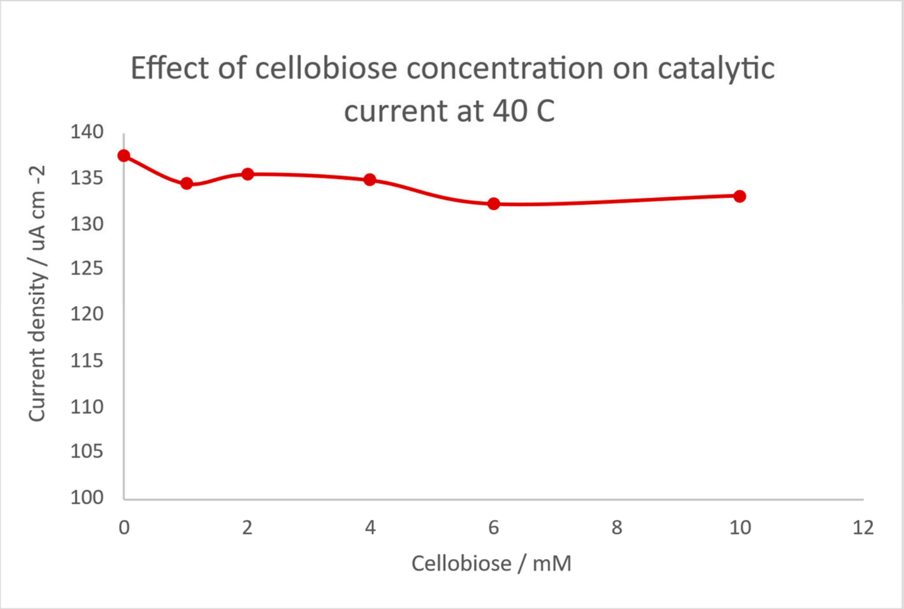

Figure S2. Effect of cellobiose concentration on the GOx bioanode. Current densities of the GOx modified bioanode from 1 mM glucose at various concentrations of cellobiose (1-10 mM) in citrate buffer pH 5.5 at the applied potential 0.28 V (vs Ag/AgCl 3 M KCl) under argon-saturated electrolyte, rotation speed 1000 rpm, temperature 40 ^{o}C.

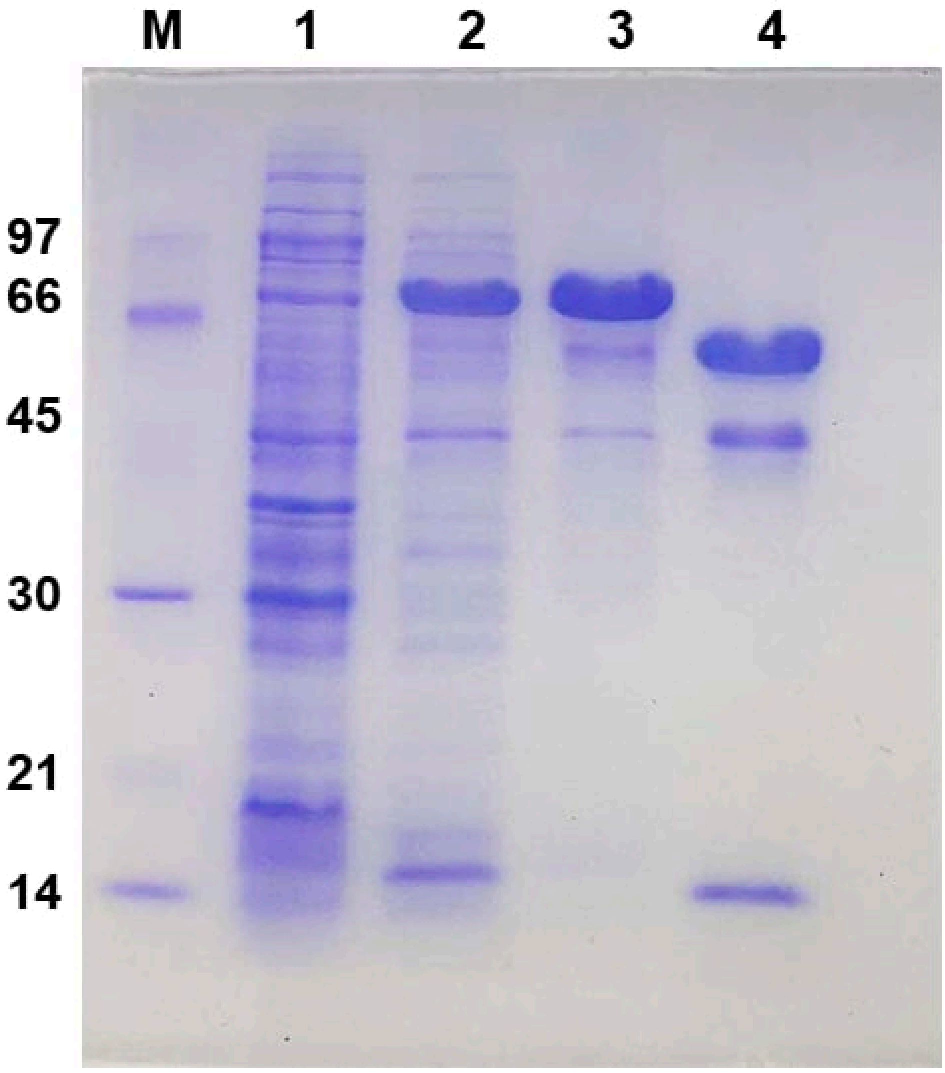

Figure S3. SDS-PAGE analysis of the purification fractions of *Th*Cel6A enzyme from recombinant expression. Molecular weight markers are on the left (marked by MW in kilodaltons), followed by lane 1, insoluble pellet; lane 2, crude soluble fraction; lane 3, purified protein after first IMAC column; lane 4, wash of the IMAC column with equilibration buffer; lane 5, wash with 5 mM imidazole; lane 6 and lane 7 wash with 10 mM imidazole; lane 8 to lane 17 wash with 250 mM imidazole and lane 18 wash with 500 mM imidazole.

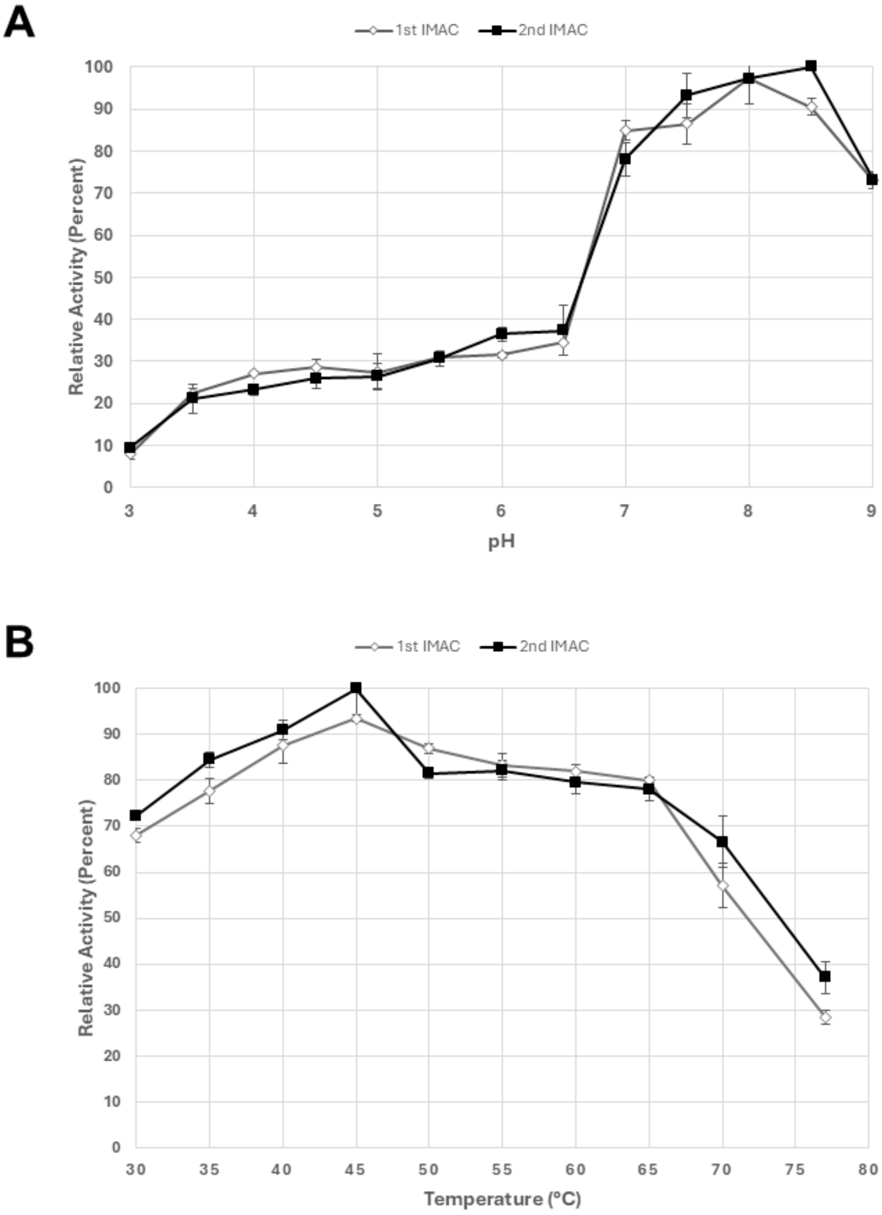

Figure S4. *Th*Cel6A endoglucanase activity dependence on pH and temperature. The optimal pH (**A)** and temperature **(B)** of *Th*Cel6A were assessed by measuring the specific activity for hydrolysis of 0.5% barley beta-glucan for protein with the fusion tag (1st IMAC) and the fusion tag removed (2nd IMAC). The pH effect was assessed at 50 °C in citrate/phosphate buffers at the designated pH and the temperature effect assessed in 50 mM Tris-HCl pH 8.5. The values are the means +/- SDs from three replicates.

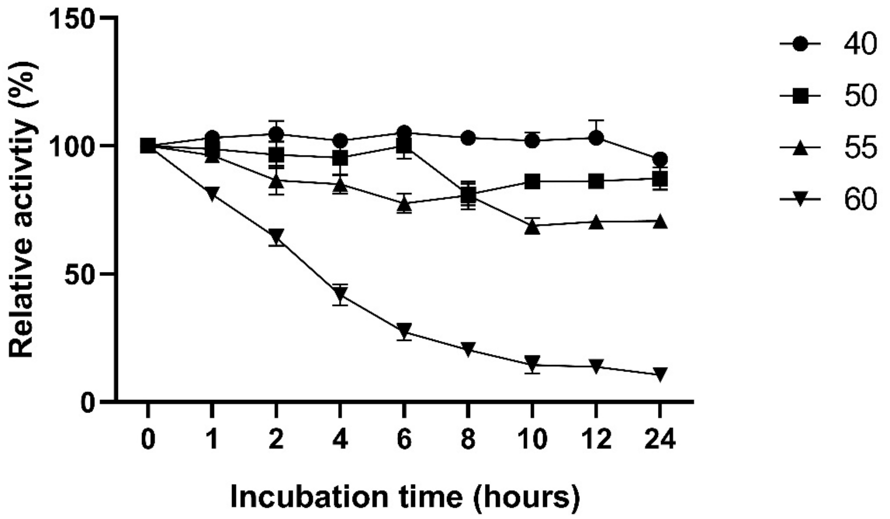

Figure S5. Thermal stability of *Th*Cel6A. The thermostability of *Th*Cel6A assessed with 0.5% CMC as the substrate. The activity of unheated sample was set as 100% activity, and the relative hydrolytic activity at each time point is presented as a percentage. The values are the means +/- SDs from three replicates.

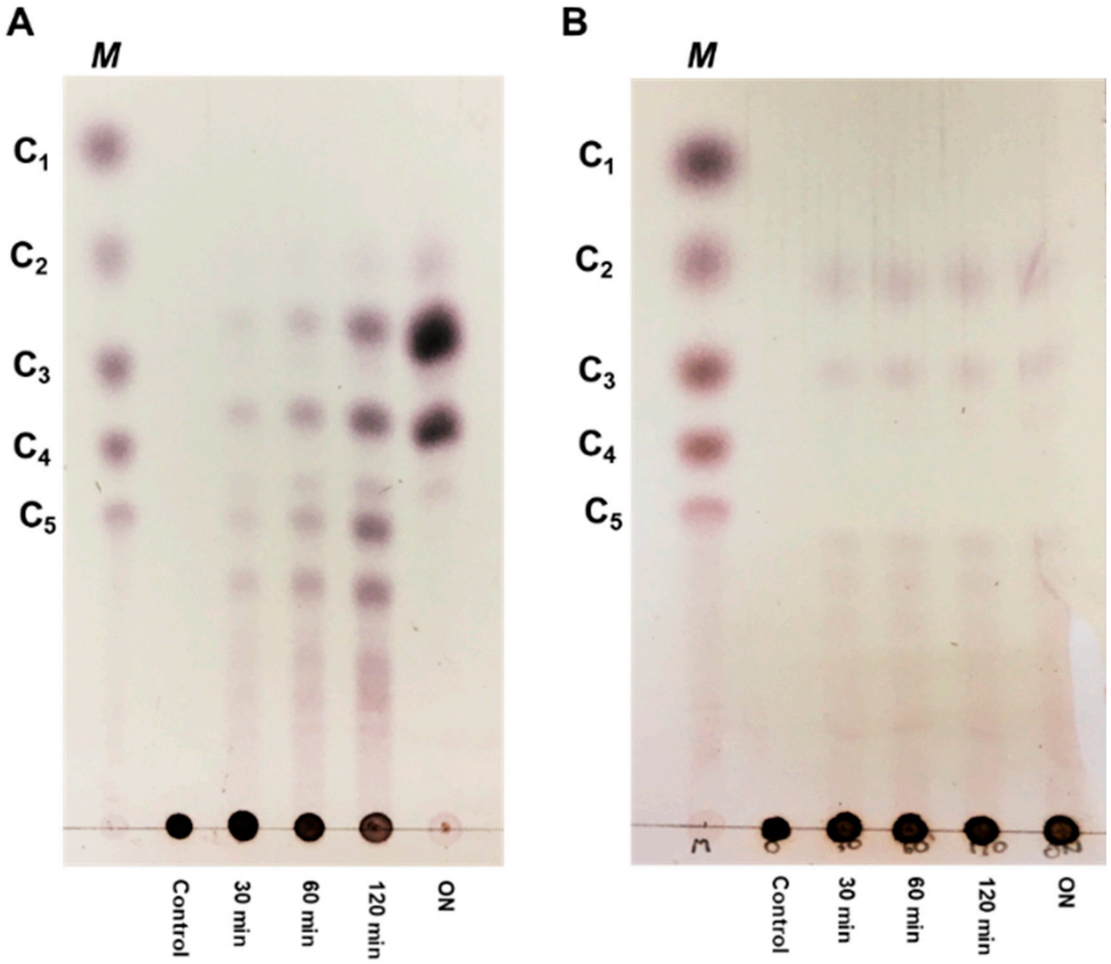

Figure S6. Thin-layer chromatography of the hydrolysis product of *Th*Cel6A against Barley β D glucan **(A)** and CMC **(B)**. The hydrolysis product profiles of *Th*Cel6A were detected after 30 min to overnight (ON) incubation at the optimal reaction condition. The glucose and cellooligosaccharide standards are marked C1-C6 representing their degree of polymerization.

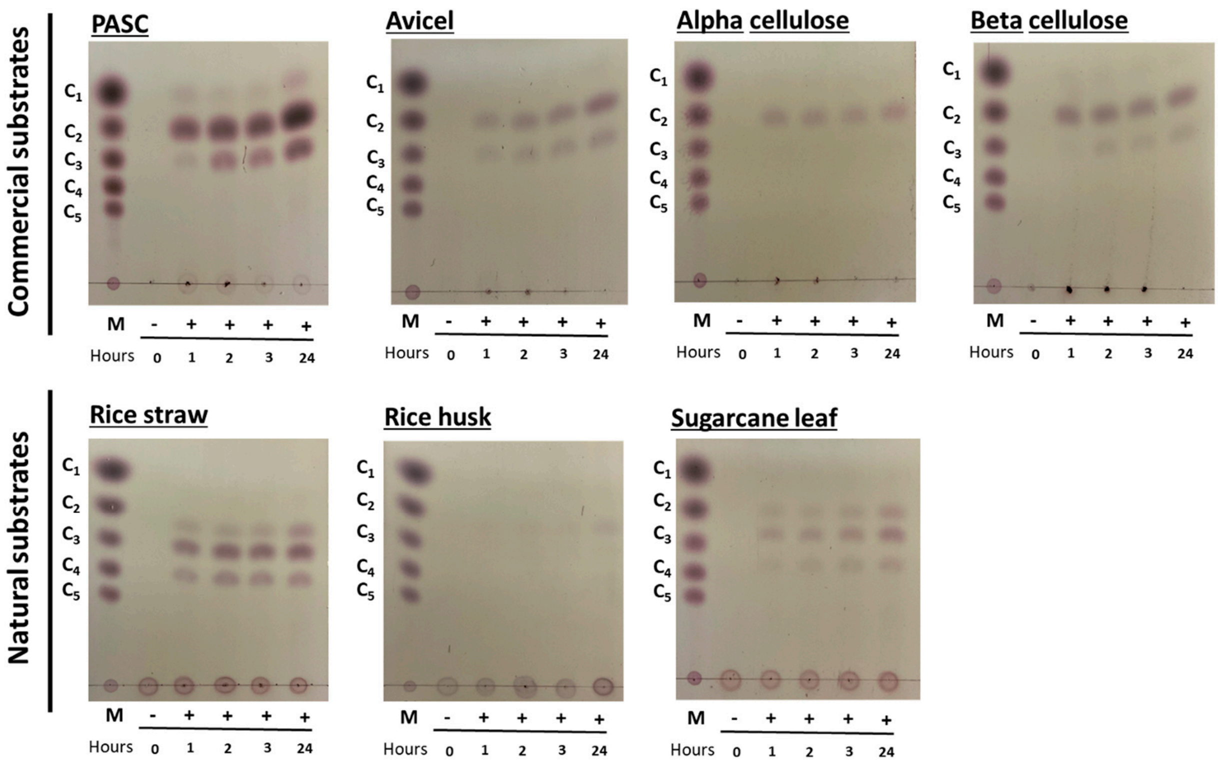

Figure S7. Thin-layer chromatography of the hydrolysis product of *Th*Cel6A against commercial and natural polysaccharide substrates with different incubation time, ranging from 1 hour to 24 hours. The soluble products of *Th*Cel6A hydrolysis of polysaccharide and biomass substrates were detected after boiling and running on silica gel TLC. Lane M, the standard mixture of glucose (C_1) and cello-oligosaccharides (C_2-C_6), for which the numbers represent the degree of polymerization. PASC is phosphoric acid-treated cellulose; all other substrates were pretreated with NaOH before hydrolysis reactions.
